# Monocyte Chemoattractant Protein-1 (MCP-1/CCL2) Drives Activation of Bone Remodelling and Skeletal Metastasis

**DOI:** 10.1007/s11914-019-00545-7

**Published:** 2019-11-11

**Authors:** Bridie S. Mulholland, Mark R. Forwood, Nigel A. Morrison

**Affiliations:** School of Medical Science and Menzies Health Institute Queensland, Griffith University, Gold Coast, Queensland 4222 Australia

**Keywords:** MCP-1 or CCL-2, Breast cancer, Osteoclast, Bone remodelling, Metastasis

## Abstract

**Purpose of Review:**

The purpose of this review is to explore the role of monocyte chemoattractant protein-1 (MCP-1 or CCL2) in the processes that underpin bone remodelling, particularly the action of osteoblasts and osteoclasts, and its role in the development and metastasis of cancers that target the bone.

**Recent Findings:**

MCP-1 is a key mediator of osteoclastogenesis, being the highest induced gene during intermittent treatment with parathyroid hormone (iPTH), but also regulates catabolic effects of continuous PTH on bone including monocyte and macrophage recruitment, osteoclast formation and bone resorption. In concert with PTH-related protein (PTHrP), MCP-1 mediates the interaction between tumour-derived factors and host-derived chemokines to promote skeletal metastasis. In breast and prostate cancers, an osteolytic cascade is driven by tumour cell–derived PTHrP that upregulates MCP-1 in osteoblastic cells. This relationship between PTHrP and osteoblastic expression of MCP-1 may drive the colonisation of disseminated breast cancer cells in the bone.

**Summary:**

There is mounting evidence to suggest a pivotal role of MCP-1 in many diseases and an important role in the establishment of comorbidities. Coupled with its role in bone remodelling and the regulation of bone turnover, there is the potential for pathological relationships between bone disorders and bone-related cancers driven by MCP-1. MCP-1’s role in bone remodelling and bone-related cancers highlights its potential as a novel anti-resorptive and anti-metastatic target.

## Introduction

Monocyte chemoattractant protein-1 (MCP-1) is a member of the CC-motif chemokine family (as CCL2); a large group of cell signalling molecules and cognate receptors. MCP-1 was the first discovered human chemokine and is well-known as a potent chemotactic factor for monocytes [1–3]. It is produced by a number of different cell types, including endothelial, epithelial, smooth muscle, mesangial, astrocytic, monocytic, microglial and fibroblastic. MCP-1 is either constitutively produced or induced subsequent to oxidative stress, specific cytokine activity or specific growth factor activity [1]. MCP-1 mediates its action through CC receptors (CCRs), predominantly CCR2. Dissimilarly to MCP-1, CCR2 is not so universally expressed, with its expression mostly restricted to vascular smooth muscle cells, mononuclear cells, monocytes and activated natural killer (NK) cells [1]. One of the peculiar aspects of CC chemokine biology is that a high degree of cross-talk exists between receptors and chemokines. Chemokines act as both homodimers and as heterodimers with structurally similar chemokines—a particular chemokine may interact with other chemokines and with several primary receptors and, if at a high enough concentration, may possibly interact with other, atypical receptors [4]. Chemokines also have affinity for extracellular matrix molecules, such as glycosaminoglycans (GAGs), which alters the effective concentration.

A further characteristic of chemokine biology is the proteolytic processing of chemokines [4], which can produce dominant-negative forms and, in some cases, more potent forms. A dominant-negative form of MCP-1 exists and is referred to as 7ND; MCP-1 with 7 amino acids truncated from the N-terminus. It completely inhibits the action of MCP-1 and has gained traction as a useful investigative tool and as a potential novel therapeutic [5•, 6, 7]. Despite such functional complexity in chemokine biochemistry, inflammatory chemokines usually elicit strong cellular responses—MCP-1 has been widely accepted as a profound inflammatory mediator, having both pro-inflammatory and anti-inflammatory roles [1]; consequently, MCP-1 has been the subject of many studies. There is mounting evidence for the involvement of MCP-1 in bone remodelling as a critical mediator, the pathogenesis of particular bone diseases and the metastasis of particular cancers to the bone; the focal point of this review.

## Bone Remodelling—the Fundamental Basis of Bone-Related Diseases

The roots of disease can be found in the physiological mechanisms that underpin an organ's normal action. Physiological bone remodelling is a critical contributor to overall health, having roles in growth, structural preservation, repair and mineral homeostasis [8–10]. Bone remodelling is a coordinated process that integrates bone resorption and bone formation by osteoclasts and osteoblasts, respectively [11, 12]. This occurs in a controlled and coupled manner and works to remove old bone and replace it with new bone. The balance of such remodelling can favour increased bone mass (anabolic) or bone loss (catabolic) outcomes. Extremes of bone remodelling result in pathology; for example, when resorption exceeds bone formation the onset of osteoporosis ensues.

Osteoblasts are specialised bone-forming cells that arise from pluripotent mesenchymal stem cells [12–14]. Osteoblasts produce bone matrix proteins, are critically involved in bone mineralisation and express factors, such as macrophage-colony stimulating factor (M-CSF) and receptor activator of nuclear factor kappa beta-ligand (RANKL) that are necessary for the differentiation and maturation of osteoclasts [12]. Some osteoblasts that are embedded in bone matrix differentiate into osteocytes, the most abundant cells in the bone that exist within the lacunae and form cell-cell networks through canaliculi [15]. Osteoclasts are multinucleated, terminally differentiated giant cells that form by fusion of cells of the monocyte-macrophage lineage and, as such, are derived from haematopoietic stem cells [13, 14]. Osteoclasts are defined in vitro as multi-nucleated cells with at least three nuclei that are capable of forming resorption pits in bone and that express a number of characteristic markers, including tartrate-resistant acid phosphatase (TRAP), cathepsin K and calcitonin receptor [12].

## Cancer’s Existence in Bone

Bone cancer presents in two unfavourable forms: primary and secondary [16]. Primary bone cancer refers to cancer that originates and is implicated within the bone microenvironment and is not to be confused with bone marrow cancer, which describes cancers that originate within the bone marrow and are implicated in the blood. Primary bone cancer is exceptionally rare, accounting for less than 0.2% of all cancers [17]. Secondary bone cancer refers to bone metastases, in which cancerous cells have disseminated from another tissue location and colonised in the bone [18]. Bone is a frequent site of metastasis, with lung, prostate and breast cancer reflecting the majority of skeletal metastases [18, 19]. Bone metastases are accompanied by a poor prognostic outlook; one that has not changed significantly in decades [20].

Metastases require intricate communication between cancerous cells and endogenous cells at the metastatic site. This interaction facilitates the dissemination of cancer cells to other locations within the body and adds a complex dimension to cancer treatment. The formation of bone metastases is controlled, in part, by the pre-metastatic and metastatic niches, in which the homing and colonisation of cancerous cells to a related tissue location occurs through the action of endogenous factors [21]. The role of these niches in metastatic cancer development is an expansion of the ‘seed and soil’ theory coined by Stephen Paget in the late 1880s [22]. He used a simple analogy to shed light on the incredible complexity of secondary tumour development; the seed will grow in soil that favours its growth—cancer will disseminate to organs that provide a suitable micro-environment to support and encourage the growth of the tumour; the pre-metastatic and metastatic niches refer to the critical, metastasis-promoting micro-environmental changes that occur to the primary tumour organ and the metastatic organ site, respectively. Thus, for cancers that preferentially metastasise to the bone, the bone micro-environment must play a critical role [23].

## The Role of MCP-1 in Bone Remodelling

The relationship of MCP-1 to bone remodelling is manifested through its involvement in osteoclast differentiation and maturation. Given a long-established role in monocyte responses to infection and injury, it was somewhat unexpected that MCP-1 had a role in osteoclast biology and, in particular, its stimulation of osteoclast formation. Since MCP-1 stimulates chemotaxis of monocytes [1], early studies on the role of MCP-1 in the bone focussed on this known chemotactic effect. Early studies from Graves et al. (1999) and Volejnikova et al. (1997) demonstrated that MCP-1 is predominantly expressed by bone-forming osteoblasts—both studies noted an increase in monocyte recruitment and associated MCP-1 with the regulation of bone remodelling [24–26].

Osteoclasts differentiate from cells of the monocyte lineage; however, osteoclast differentiation requires the presence of M-CSF and RANKL: M-CSF is required for precursor cell survival while differentiation itself is controlled by RANKL [8]. Monocyte-like precursors can differentiate into other cell types—the decision on which cell type is mediated by the presence and concentration of other signals. Notably, granulocyte macrophage-colony stimulating factor (GM-CSF) suppresses human osteoclast differentiation in vitro, even in the presence of RANKL and M-CSF [27••]. Interestingly, this complete suppression of osteoclast formation by GM-CSF has been strongly reversed by addition of MCP-1 [27••]. Similarly, treatment with an anti-MCP-1 antibody strongly suppresses human osteoclast formation in vitro [27••, 28], with similar suppression of osteoclast formation observed using 7ND, a dominant negative form of MCP-1 with truncation of seven amino acids [5•]. This mutant forms inactive heterodimers and inhibits the protein’s action [29].

The interaction between osteoblasts and osteoclasts with respect to RANKL and M-CSF is well known; the interaction between osteoblasts and osteoclast progenitors is not. Early work showed that recruitment of monocytes to the bone surface is mediated by MCP-1 [30]. Li et al. (2007) have since shown that parathyroid hormone (PTH) is capable of inducing expression of MCP-1 by osteoblasts and mediating this recruitment of monocytes [31••]. Further, MCP-1 is specifically induced in bone by stress fracture (SFx) initiation, activating targeted remodelling of the SFx [32], and is expressed by osteoblasts and osteocytes at the periosteal exit point of the SFx [33]. In addition to mature osteoblasts, this MCP-1-mediated interaction may start earlier in the osteoclast lineage. Using the RAW264 cell line as a model for osteoclast progenitors, Sumi et al. (2018) showed that mesenchymal stem cells produce MCP-1 as a chemotactic signal for osteoclast progenitors via MCP-1’s receptor, CCR2 [34]. Subsequently, MCP-1 has been recognised as a regulator of osteoclast biology as a pro-survival and pro-differentiation agent.

**Fig. 1:**
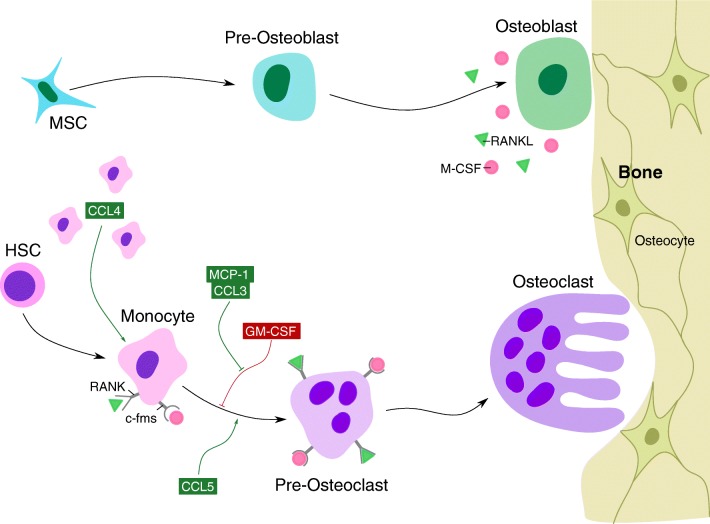
Role of notable chemokines in osteoclastogenesis. Osteoclast differentiation is primarily controlled by M-CSF and RANKL expression—M-CSF and RANKL expressed by osteoblasts bind their respective receptors c-fms and RANK on osteoclast precursors, driving monocyte differentiation into osteoclasts. A number of chemokines play an integral role in ensuring the success of osteoclastogenesis, notably CCL4, MCP-1, CCL3 and CCL5. CCL4 is responsible for the recruitment of osteoclast precursors; CCL3 and MCP-1 both reverse and rescue osteoclast differentiation from GM-CSF repression; and CCL5 strongly promotes osteoclast formation

MCP-1 is rapidly induced in human osteoclast progenitors when differentiating into osteoclasts. This induction of MCP-1 precedes a similar but weaker induction of chemokine (C-C) motif ligand-3 (CCL3) [5•]. Like MCP-1, CCL3 is capable of reversing the suppression of osteoclast formation by GM-CSF [35]. Consistent with the action of its receptor, CCR5, which is a regulator of osteoclast formation and osteoporotic bone loss [36], CCL5 (RANTES) also strongly promotes human osteoclast formation and is able to do so in the presence of inhibiting levels of a neutralising MCP-1 antibody [27••]. In contrast to MCP-1, which fully reversed GM-CSF repression of osteoclast formation, RANTES recovered multinuclear cell formation, but the cells lacked the ability to resorb bone [27••]. Consistent with the expression of its receptor, and another potential receptor of MCP-1, CCR4 in human osteoclast precursors [35], CCL4 (also known as MIP1b) does not enhance osteoclast formation but was found to be chemotactic to osteoclast precursors [37]. Therefore, several chemokines of the CC family (MCP-1, CCL3 and CCL5) are produced in differentiating human osteoclasts and all have similar actions in promoting osteoclast fusion and survival. It is yet to be determined how multiple chemokines can generate similar biological effects on human osteoclasts while maintaining some specificity of function. Although speculation, it may be that chemokines act at different time points in the differentiation process with MCP-1, at least, being very early and strongly induced in human osteoclast precursors (Fig. 1) [5•].

## MCP-1 Knock-Out Models for Determination of its Role in Bone Remodelling

The literature shows clear involvement of MCP-1 in bone remodelling, specifically in osteoclast differentiation; however, its exact role in this process remains to be determined. As mentioned, the primary receptor for MCP-1 is CCR2, which is expressed in human osteoclast precursors [35] and exists in humans in two isoforms: CCR2A and CCR2B. Mononuclear cells and vascular smooth muscle cells express the CCR2A isoform while monocytes and activated NK cells express the CCR2B isoform [1, 38]. The presence of these two isoforms lends the possibility of the activation of different pathways eventuating in different effects. Consequently, CCR2 has been identified as a dual-action receptor that is implicated in both pro-inflammatory and anti-inflammatory pathways [1]. Studies on the bone phenotype of the CCR2 knockout (-/-) mouse [39•] are remarkably similar to those recently observed in the CCR5-/- mouse [36]. Lee et al. (2017) found CCR5-/- mice to be resistant to RANKL-induced bone loss. When administered with soluble RANKL (sRANKL) to induce bone loss, wild-type mice had significant reductions in bone mineral density (BMD), trabeculae number (Tb. N) and bone volume/total volume (BV/TV); conversely, when treated with sRANKL, CCR5-/- mice saw no change in the aforementioned parameters. Both CCR2-/- and CCR5-/- knockouts resulted in osteoclasts that were abundant but were defective, and, in both cases, the knockout had reduced bone loss at a level considered to be “resistant to osteoporosis” [36, 39•].

Binder et al. (2009) reported that CCR2-/- mice had a small but significant increase in tibial trabecular bone volume and a reduced number of osteoclasts in the bone at 10 weeks of age. Similarly, Sul et al. (2009) found a small but statistically significant increase in trabecular bone volume at the distal femur at 14 weeks of age. In vitro osteoclast formation assays showed that MCP-1-/- mice produced fewer osteoclasts that were about one-half as effective at bone resorption as wild type osteoclasts. Using mouse cells, Sul et al. (2012) confirmed earlier observations seen in human cells [27••]; that addition of exogenous MCP-1 boosted osteoclast numbers in the in vitro cultures and significantly improved bone resorption, and that anti-MCP-1 neutralising antibody inhibited osteoclast formation [40]. MCP-1 gene induction is an early event in osteoclastogenesis. Sul et al. (2012) saw a 29-fold induction of MCP-1 mRNA within the first 8 h in mouse bone marrow cells using pure human colony forming unit-granulocyte macrophage (CFU-GM), and Morrison et al. (2014) saw more than a 1000-fold change in MCP-1 transcript at 24 h [5•, 40]. Taken together, these experiments show that MCP-1 is a potent paracrine and autocrine agent acting to promote the formation of osteoclasts and, therefore, is capable of influencing bone loss in oestrogen-withdrawal situations, such as in osteoporosis, and inflammatory situations, such as in osteo- and rheumatoid arthritis.

## The Role of MCP-1 in PTH Regulation of Bone Remodelling

In addition to having a role in the cells that underpin bone function, MCP-1 also has a role in the hormonal regulation of bone. Decreased serum calcium activates the parathyroid gland to secrete PTH, increasing osteoclast activity to release calcium from bone and normalise serum calcium levels [11]. Excessive bone loss occurs in conditions with continuously elevated PTH (cPTH), such as primary hyperparathyroidism, where cPTH creates a catabolic effect that increases osteoclast number, eventuating in the onset of osteoporosis [11]. Conversely, intermittent PTH (iPTH) creates a potent anabolic effect on bone, which forms the basis of an effective osteoporosis therapy. The anabolic effect of iPTH has been the subject of many recent reviews.

Remarkably, MCP-1 is the most iPTH-responsive gene with > 200-fold induction in bone after 14 days of iPTH treatment [31••]. Both osteocytes and osteoblasts are positive for MCP-1 protein after iPTH [41]. Other notable target genes follow the pattern of MCP-1. RANKL expression pulses after iPTH injection, with about a 20-fold change (Morrison NA, unpublished). MCP-1 has a strong stimulatory effect on osteoclast formation and bone resorption [5•, 27••, 31••, 35, 42••] and so may work with RANKL in pulses to increase osteoclast recruitment and activity.

It now appears, though, that MCP-1 is involved in regulating both the anabolic and catabolic actions of PTH via osteoclast formation [43]. An osteoclast formation defect in MCP-1 null mice was observed by Sul et al (2012) who did not study PTH effects, but rather examined osteoclast differentiation [40]. A key observation by Siddiqui et al. (2017) highlights the functional importance of MCP-1 as a key bone regulator [44]. Continuous PTH treatment (catabolic) stimulates osteoclast formation via increased RANKL expression and is considered an adequate explanation of the increase in osteoclast numbers during PTH treatment. In the MCP-1-/- animal, continuous PTH treatment caused exactly the same rise in RANKL expression as in wild type. Furthermore, regulation of osteoprotegerin (OPG) was virtually identical in the MCP-1-/- animal as in the wild type [44]. These data support the surprising fact that, after PTH treatment, osteoclast deficiency in the MCP-1-/- animal occurs in the presence of normally induced RANKL expression levels that are entirely adequate in the wild type. In other words, MCP-1 expression levels are an important consideration, as well as the RANKL/OPG ratio.

## MCP-1 and Bone-Related Disorders in the Clinical Setting

Diseases of the bone characteristically include some type of interruption, interference or inhibition of the processes associated with bone remodelling. Bone loss induced by inflammatory diseases is believed to occur as a result of direct or indirect effects of inflammatory cytokines or inflammatory cytokine networks [45]. Rheumatoid arthritis (RA) and osteoarthritis (OA) are the most common forms of arthritis [46] and represent a significant threat to the healthy ageing of the global community [47].

Patients suffering from RA have significantly higher serum concentration levels of MCP-1 compared to control patients, with this increase in serum concentration levels also positively correlated with the number of osteoclasts cultured from their peripheral blood monocytes (PBMCs) [48]. Recently, MCP-1 was positively associated with the presence and progression of knee OA [49]. In adjusted logistic models, each unit increase in log[MCP-1] amounted to a 75% increase in the incidence of knee OA at the 5-year follow-up mark, with implications in the presence and progression of knee OA and medial joint space narrowing, but not with the presence or progression of osteophytes, bone sclerosis, knee symptoms or symptomatic knee OA [49].

One commonly used treatment for late-stage arthritis is the total joint replacement (TJR) [6]. In cases of severe arthritis, TJRs are an outstanding method to relieve obstinate joint pain and improve joint function [50]. One setback of TJRs is the production of particulate debris, referred to as wear particles, that begins at implantation and continues throughout the lifespan of the prosthesis [50]. TJR failure is typically caused by wear particle–induced periprosthetic osteolysis and the implant loosening that occurs as a result [50]. Local delivery of 7ND into the space between the calvarial bone and periosteum significantly decreases wear particle–induced osteolysis [6]. This indicates an influential role of MCP-1 in the mechanism of wear particle–induced osteolysis and identifies it as a potential target for mitigating this undesirable side effect of TJRs [6]. An adaptation of this method saw the local delivery of MCP-1 by 7ND-coated titanium rods, and also noted a decrease in systemic macrophage recruitment, osteoclast number and wear particle–induced osteolysis [7].

Undoubtedly, one of the most readily associated diseases with bone is osteoporosis. Osteoporosis is a condition characterised by the disruption to the critical balance between bone resorption and bone formation, in which a favouring of bone resorption leaves the skeleton in a state of disharmonic predilection to bone loss [39•, 51]. This disease is well studied and typically associated with the overactivity of osteoclasts [39•]; subsequently, MCP-1 is implicated in the disease pathogenesis through its role in osteoclastogenesis. Furthermore, MCP-1 levels are increased in post-menopausal women when compared to controls [52]. These elevated MCP-1 levels are also associated with a decrease in bone mineral density and attenuated oestrogen concentrations, and positively associated with inflammatory markers tumour necrosis factor-α (TNFα), interleukin-6 (IL-6) and visual analogue scores, a pain assessment used with post-menopausal osteoporosis sufferers, potentially highlighting MCP-1 as a biomarker for reflecting post-menopausal osteoporosis disease severity [52].

## MCP-1 and its Relationship to Skeletal Metastasis

MCP-1 is implicated in a number of cancers and, coupled with its involvement in bone remodelling, places it in a central position to link these roles with bone metastasis [53]. This also endows MCP-1 with some potential as a novel therapeutic target. In recent years, preclinical studies have proposed selective and non-selective MCP-1 antagonists as effective therapeutic agents for a range of inflammatory diseases; however, its therapeutic potential against tumour progression has less evidence. While studies are accumulating, their outcomes for MCP-1 inhibition for the treatment and prevention of metastatic cancers remain inconclusive [38]. With associations to poor prognosis and adverse clinical outcomes, metastasis is the chief obstacle preventing the successful clinical management of cancer. One of the best representations of the negative impact on cancer prognosis that metastases have is the notable decrease in 5-year survival rates in prostate and breast cancer [54].

The formation of bone metastases is controlled, in part, by the pre-metastatic and metastatic niches [21]. It has been established that the chemokine, stromal-derived factor-1 (SDF-1 or CXCL12), is implicated in homing of tumour cells to bone [55], where metastatic breast cancer cells, for example, express its receptor CXCR4 [56]. Tumour-induced osteolysis occurs through osteoclast appropriation. That is, the recruitment of osteoclasts by cancer cells is a central feature of the bone tumour metastatic niche [57]. Osteoclastogenesis and bone resorption are also independent, but pivotal steps in the formation of skeletal metastases [58]. Bone resorption, for example, has been associated with amplification of metastatic osteolysis by releasing transforming growth factor beta (TGFb) from the matrix [59•].

Since the early 90s, PTHrP derived from cancer cells has been recognised as a key signal driving metastatic osteolysis [59•]. PTHrP, secreted by cancer cells, acts like PTH through PTH1R to increase RANKL expression by osteoblastic cells in the local environment, promoting osteoclastogenesis. But like PTH, PTHrP also upregulates MCP-1 expression with similar potency [60] and stimulates anabolic effects in bone [61]. This combined effect of PTHrP provides a potent stimulus to both osteoclast recruitment and differentiation in the metastatic niche. In prostate cancer, an osteolytic cascade is driven by tumour cell–derived PTHrP that stimulates induction of MCP-1 by osteoblastic cells in the bone marrow niche [62••]. The MCP-1, in turn, acts in an autocrine manner through CCR2 on prostate tumour cells, stimulating the release of vascular endothelial growth factor-A (VEGF-A). These events accelerate tumour growth via enhanced osteoclastic and endothelial cell activity in the bone marrow, supported by increased angiogenesis [62••]. Hence, MCP-1 mediates the interaction between tumour-derived factors and host-derived chemokines acting in cooperation to promote skeletal metastasis.

With similar importance to osteoclast recruitment is tumour-associated macrophage (TAM) recruitment. The role of TAMs in tumour progression is varied and occurs at different levels; from the promotion of genetic instability to the suppression of protective adaptive immunity. TAMs have important roles across all stages of tumourigenesis, encompassing cancer stem cell nurturing and metastasis promotion [63]. While their importance in tumourigenesis is understood, their exact mechanism has not been described in detail [58]. For example, zoledronic acid (ZA) is an effective additive therapy in the management of early stage breast cancer. It prevents tumour-promoting effects of mesenchymal stem cells by impairing TAM recruitment via decreased MCP-1 expression [64].

MCP-1 expression by tumours is correlated to the number of TAMs. Tewari et al. (2016) reported that elevated MCP-1 levels were significantly associated with decreased breast cancer survival, and MCP-1, along with NF-κB and TAMs, was identified as a key determinant of poor prognosis and low survival; highlighting its potential as a prognostic marker [65]. MCP-1 also drives the recruitment of myeloid cells to triple-negative breast cancers [66]. Its high expression in breast cancer–associated macrophages, and in the stroma, is associated with poor prognosis in basal-like breast cancer [67]. In a co-graft tumour model, fibroblast-derived MCP-1 attenuated the effect of an MCP-1-neutralising antibody, while mutation of CCR2 in cancer cells significantly reduced tumour growth, indicating MCP-1 involvement in basal-like cell cancer progression [67]. With its extensive implications in breast cancer progression, MCP-1 targeted monotherapy presents itself as a useful therapeutic. Bonapace et al. (2014) found, nonetheless, that cessation of MCP-1 inhibition resulted in a rapid increase in metastasis and accelerated death. They cautioned the appropriateness of MCP-1 targeted monotherapy for breast cancer, highlighting a potentially dangerous flaw in this novel therapeutic [68].

MCP-1, along with a manner of other inflammatory proteins, has been noted in many papers as an important component of cancer progression. Table 1 describes aspects of bone metastasis formation where MCP-1 has been demonstrated to have a dominant role.

**Table 1:** The role of MCP-1 in the metastasis of different cancers to bone.

Type of cancer	MCP-1 involvement	Reference
Prostate	Docetaxel increased MCP-1 expression and this increase in expression resulted in a protective effect, decreasing the chemotherapeutic effectiveness of Docetaxel	[69]
	MCP-1 expression by adipocytes in obese individuals associated with prostate cancer progression and invasiveness	[70]
Lung	MCP-1 implicated in the promotion of CCR2^+^/Ly6C^hi^ monocytes and the induction of CCR2^+^ endothelial cell vascular permeability in the lungs	[71]
	Fibrocytes elicit a pre-metastatic niche-conditioning effect through the recruitment Ly6C^+^ monocytes via MCP-1	[72]
	MCP-1 positively associated with metastasis-related bone loss	[73]
Nasopharyngeal	Expression of MCP-1 higher in poorly differentiated NPC cells compared to highly differentiated NPC cells, suggesting that MCP-1 plays a role in the maturation and progression of these cancerous cells	[74]
Multiple myeloma	MCP-1 is over-expressed – MCP-1 expression in bone marrow stromal cells is upregulated by human myeloma cells	[75]
Oral squamous cell carcinoma	Associated with elevated expression of MCP-1	[42••]
	Inhibition of MCP-1 with its dominant-negative form, 7ND, successfully inhibited osteoclast differentiation and limited the capacity of OSCC to invade surrounding bone	[76]
Myeloid leukaemia	Implicated in cell transmigration and proliferation; however, not in chemotherapy resistance	[77]

These studies present a convincing case for the involvement of MCP-1 in skeletal metastasis. In a model of breast cancer bone metastasis, Ottewell et al. (2014) introduced the probability of a causal relationship between post-menopausal osteoporosis and the colonisation of disseminated breast tumour cells in bone, noting a significant difference in bone tumour occurrence in post-menopausal and pre-menopausal animals, where 83% of ovariectomised animals exhibited cancerous bone lesions compared to 17% of sham animals [78••].

Wright et al. (2017) expanded on this concept, investigating the potentially negative effects that some pharmaceuticals may have through activation of bone resorption. They specifically examined aromatase inhibitors (AI), a popular anti-cancer drug used in the treatment of breast cancers in post-menopausal women. They observed that prevention of AI-induced bone resorption mitigated the formation of bone metastases; subsequently implicating this treatment-induced bone loss in metastatic breast cancer progression [79].

**Fig. 2:**
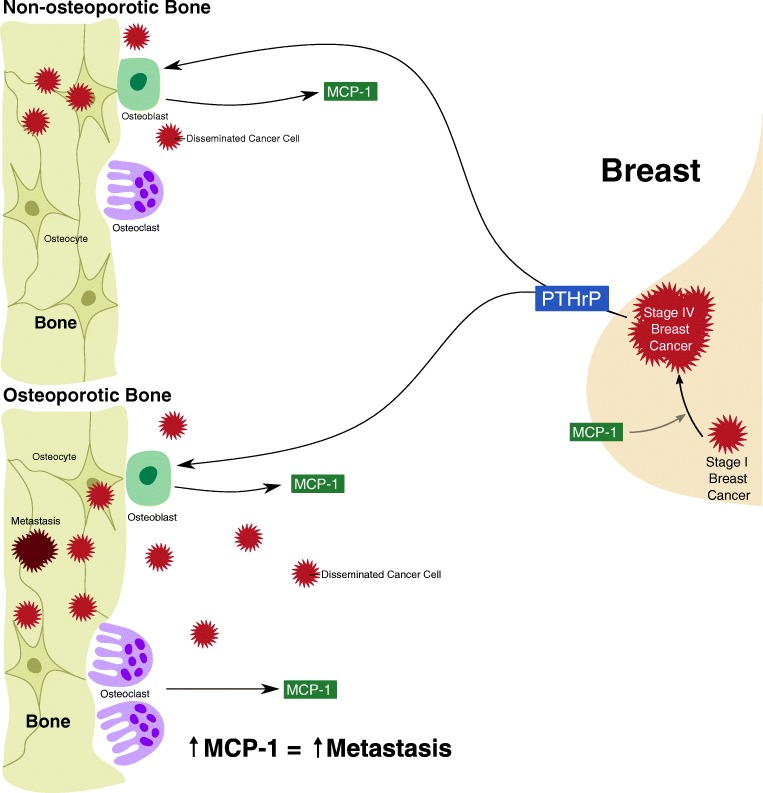
Potential role of osteoporosis and MCP-1 in breast cancer bone metastasis. PTHrP plays a pivotal role in breast cancer progression and osteoblastic expression of MCP-1. This relationship between PTHrP and osteoblastic expression of MCP-1 related to cancer progression may drive the colonisation of disseminated breast cancer cells in the bone. The increased osteoclast activity associated with osteoporosis results in an increase in MCP-1 expression. This further increase in MCP-1 expression in osteoporotic bone may increase the rate and likelihood of bone metastasis following breast cancer. MCP-1 is also involved in breast cancer progression, and so forms a vicious cycle of MCP-1 and PTHrP expression in progressing breast cancer, and MCP-1 expression in bone that encourages bone metastasis

Breast cancer frequently eventuates in bone metastases [80]. The role of MCP-1 in breast cancer bone metastasis is less clear than its role in the bone metastatic progression of other cancers. MCP-1 has been widely implicated in the metastasis of breast cancer to the lung—Qian et al. (2011) showed that MCP-1 promoted breast cancer lung metastasis by recruiting inflammatory monocytes, and Bonapace et al. (2014) showed an acceleration of breast cancer lung metastasis subsequent to the cessation of MCP-1 inhibition [3, 68]. Takahashi et al. (2009) proposed an opposing role of MCP-1, concluding that MCP-1 negatively regulates breast cancer metastasis to the bone and lung, and associated MCP-1 inhibition with a greater incidence of breast cancer bone and lung metastasis in their murine model [81]. Nevertheless, given its role in osteoclastogenesis, osteoporosis-related bone loss and skeletal metastasis, MCP-1 may be critically involved in the relationship between osteoporosis, breast cancer and bone metastasis (Fig. 2).

The formation of metastases is only possible because of the intricate communication between disseminated cancer cells and the metastatic organ of choice. Cancer cells communicate best with organs that have similar microenvironmental properties as the organ in which they originated. MCP-1 is ever-present within the breast tumour microenvironment as it drives breast cancer progression; similarly, MCP-1 is ever-present within the bone as a mediator of bone remodelling, and even more so within osteoporotic bone, given the increased osteoclastic expression of the chemokine. These similarities in MCP-1 expression may facilitate the communication between breast cancer cells and the tumour microenvironment, presenting MCP-1 as a driving factor of breast cancer bone metastasis.

## Conclusion

Advances in cancer biology have highlighted the inherent need for, and the great advantage of, targeted therapies. There is mounting evidence to suggest a pivotal role of MCP-1 in the mechanisms that underpin bone remodelling and skeletal metastasis. Evidence suggests a strong tumourigenic role for MCP-1 in cancer through its effects on monocyte recruitment, activation of tumour-associated macrophages, induction of angiogenesis, metastasis promotion and osteoclastogenesis. In breast and prostate cancers, an osteolytic cascade is driven by tumour cell–derived PTHrP that upregulates MCP-1 in osteoblastic cells. This accelerates tumour growth via enhanced osteoclastic and endothelial cell activity in bone marrow, supported by increased angiogenesis. Presently, MCP-1’s specific role in breast cancer bone metastasis, as to whether it promotes bone metastases or protects the bone from breast cancer cell invasion, remains inconclusive; however, its pivotal role in bone remodelling and breast cancer progression highlights the need for further investigation. Development of successful treatments for metastatic cancers proves challenging, especially for skeletal metastases where surgery to excise tumours is often impossible. Targeting MCP-1 has promise as a therapeutic option with increasing evidence for its role as a primary contributor to the development of several types of metastatic lesions; however, the reported metastatic rebound following anti-MCP-1 therapy in experimental animals suggests this pursuit must be navigated thoroughly and cautiously.
